# Effects of magnesium picolinate, zinc picolinate, and selenomethionine co-supplementation on reproductive hormones, and glucose and lipid metabolism-related protein expressions in male rats fed a high-fat diet

**DOI:** 10.1016/j.fochms.2022.100081

**Published:** 2022-01-27

**Authors:** Kazim Sahin, Cemal Orhan, Osman Kucuk, Mehmet Tuzcu, Nurhan Sahin, Ibrahim H. Ozercan, Sarah Sylla, Sara P. Ojalvo, James R. Komorowski

**Affiliations:** a Department of Animal Nutrition, Faculty of Veterinary Medicine, Firat University, Elazig, Turkey; b Department of Animal Nutrition, School of Veterinary Medicine, Erciyes University, 38039 Kayseri, Turkey; c Department of Biology, Faculty of Science, Firat University, Elazig, Turkey; d Department of Pathology, School of Medicine, Firat University, 23119 Elazig, Turkey; e Research and Development, Nutrition 21, Harrison, NY 10577, USA

**Keywords:** Magnesium picolinate, Zinc picolinate, Selenomethionine, High-fat diet, Rat

## Abstract

This study aimed to examine the impacts of the magnesium picolinate (MgPic), zinc picolinate (ZnPic), and selenomethionine (SeMet) alone or as a combination on blood metabolites, oxidative enzymes, reproductive hormones, and glucose and lipid metabolism-related protein expressions in Wistar rats fed a high-fed diet (HFD). The rats were fed either a control, HFD, or HFD supplemented with a single (MgPic, ZnPic, SeMet) or two or three organic mineral combinations. Body weights, visceral fat, serum glucose, insulin, total cholesterol, triglycerides, leptin, malondialdehyde (MDA) concentrations as well as liver sterol regulatory element-binding protein-1c (SREBP-1c), liver X receptor alpha (LXRα), ATP citrate lyase (ACLY), fatty acid synthase (FAS), and nuclear factor kappa B (NF-κB) levels increased, while serum testosterone, follicle-stimulating hormone (FSH), luteinizing hormone (LH), sex hormone-binding globulin (SHBG), and insulin-like growth factor (IGF-1) concentrations along with liver nuclear factor erythroid 2-related factor 2 (Nrf2) levels declined in HFD rats (*P* < 0.05). Supplementing each organic mineral, but particularly the combination of HFD + MgPic + ZnPic + SeMet reversed the responses with various degrees. None of the organic elements alone or as a combination of two exerted a prominent effect on parameters measured. Although not additive or synergistic, the combination of all organic minerals added to HFD (HFD + MgPic + ZnPic + SeMet) provided the greatest responses.

## Highlights

• A high-fat diet intake leads to metabolic disorders such as obesity and diabetes.
• Dietary supplementation of Mg, Zn, and Se can be used for the alleviation of te metabolic disorder.
• A combination of MgPic, ZnPic, and SeMet is recommended for protective effects in obesity-related problems.

## 1 Introduction

Feeding a high-fat diet (HFD) results in increased body weight and fat accumulation (Maejima, Yokota, Horita, & Shimomura, 2020), which eventually leads to metabolic disorders such as obesity and diabetes (Kebede and Attie, 2014, Vernon et al., 2011). Treatment of such metabolic disorders requires altering lifestyle changes, including dietary advice and exercise. Stimulating energy metabolism aims to treat metabolic problems (Lee, Kwon, & Choi, 2018). In this respect, dietary supplementation of critical nutrients such as magnesium (Mg), zinc (Zn), and selenium (Se) can be used for this purpose.

As a cofactor in about 300 enzymes, Mg plays an essential role in carbohydrate metabolism in which Mg improves insulin sensitivity in normomagnesemic, overweight, or non-diabetic subjects (Hruby, Meigs, O’Donnell, Jacques, & McKeown, 2014). Similarly, Zn deficiency has been recognized as a risk factor for obesity and diabetes (Fukunaka & Fujitani, 2018). Although Se has a strong antioxidant feature, its effects on diabetes and obesity have been inconclusive (Tinkov et al., 2020, Vinceti et al., 2015, Wang et al., 2017).

Excessive oxidative stress and inflammation are typical indices in several chronic conditions, including obesity and diabetes (Ndisang, Rastogi, & Vannacci, 2014). Hepatic de novo lipogenesis has been increased in obesity, contributing to excessive fat mass in the body (Diraison, Dusserre, Vidal, Sothier, & Beylot, 2002). Increased oxidative stress was also linked to obesity and diabetes with reduced fertility potential in humans (Abbasihormozi et al., 2019). Various forms of inorganic sources of Mg, Zn, and Se have been used as alone or co-supplements in exploring their metabolic effects in rats, mice, and humans (Hamedifard et al., 2020, Ige et al., 2018, Qi et al., 2020, Thoen et al., 2019, Zhang et al., 2018). However, no comprehensive study has investigated the impact of the combination of magnesium picolinate (MgPic), zinc picolinate (ZincPic), and selenomethionine (SeMet) on blood parameters, male reproductive hormones, antioxidant status, and glucose and lipid metabolism-related protein expressions in male rats fed a high-fat diet. Therefore, we hypothesized that the combinations of dietary MgPic, ZincPic, and SeMet positively alter body weight changes, amounts of visceral fat, blood metabolites including glucose, insulin, triglycerides, total cholesterol, leptin, antioxidative enzyme activities, male reproductive hormones including testosterone, follicle-stimulating hormone (FSH), luteinizing hormone (LH), sex hormone-binding globulin (SHBG), and insulin-like growth factor 1 (IGF-1) concentrations as well as glucose and lipid metabolism-related protein expressions, namely liver sterol regulatory element-binding protein 1 (SREBP-1c), liver X receptor alpha (LXRα), ATP citrate lyase (ACLY), fatty acid synthase (FAS), nuclear factor kappa-light-chain-enhancer of activated B cells (NF-κB), and the nuclear factor erythroid 2-related factor 2 (Nrf2) in male Wistar rats fed an HFD.

## 2 Material and methods

### 2.1 Animals

Fifty-six male Wistar albino rats (8 weeks old) with body weights of 180 ± 20 g were obtained from the Firat University (Elazig, Turkey). The animals were housed in suitable temperature environments (22 ± 2 °C, 55 ± 5% humidity, and 12-h light/dark cycle). In the study, which was approved by the Fırat University Animal Experiments Ethics Committee (2018/19-31), all procedures were carried out according to the Animal Welfare Law and the guidelines.

Following a 7-day acclimatization period, animals were randomly divided based on equal body weights, into eight treatment groups, seven rats each, and fed either; 1) Control; rats fed a standard diet (7% fat from soybean oil wt/wt), 2) HFD; rats fed with the high-fat diet (40% fat from tallow wt/wt; Table 1), 3) HFD + MgPic; rats fed with HFD supplemented with magnesium picolinate (MgPic, 58.44 mg elemental Mg /kg diet), 4) HFD + ZnPic; rats fed with HFD supplemented with zinc picolinate (ZnPic, 17.53 mg elemental Zn/kg of diet), 5) HFD + SeMet; rats fed with HFD supplemented with selenium methionine (SeMet, 58.44 µg elemental Se/kg of diet), 6) HFD + MgPic + ZnPic; rats fed with HFD supplemented with MgPic and ZnPic, 7) HFD + MgPic + SeMet; rats fed with HFD supplemented with MgPic and SeMet, or 8) HDF + MgPic + ZnPic + SeMet; rats fed with HFD supplemented with MgPic, and ZnPic as well as SeMet. The combination treatments contained the same amounts of organic minerals as in single treatments. The standard (control) and high-fat diets (Table 1) had 550.8 ± 33.8 and 496.5 ± 48.6 mg magnesium, 29.6 ± 3.6 and 27.9 ± 2.4 mg zinc, and 0.178 ± 0.08 and 0.161 ± 0.06 mg Se per kilogram, respectively.

**Table 1:** Composition of diets (g/kg diet) fed to rats*.

	Control	HFD
Casein	200.0	200.0
Starch	579.5	150.0
Sucrose	50.0	149.5
Soybean oil	70.0	–
Beef tallow	–	400.0
Cellulose	50.0	50.0
Mineral premix**	30.0	30.0
Vitamin premix***	15.0	15.0
l-cysteine	3.0	3.0
Choline bitartrate	2.5	2.5

**Mineral Premix (g/kg mixture): anhydrous calcium carbonate 357, monobasic potassium phosphate 196, sodium chloride 74, potassium sulfate 46.6, potassium citrate monohydrate 70.78, ferric citrate 6.06, zinc carbonate 1.65, manganous carbonate 0.63, cupric carbonate 0.30, potassium iodate 0.01, anhydrous sodium selenate 0.01025.

*** Vitamin Premix (g/kg mixture): Niacin 3, Ca-pantothenate 1.6, pyridoxine-HCl 0.7, thiamine HCl 0.6, riboflavin 0.6, folic acid 0.2, d-Biotin 0.02, vitamin B12 (0.1% cyanocobalamin in mannitol) 2.5, vitamin E (all-*rac*-α-tocopheryl acetate, 500 IU/g) 15, vitamin A (all-*trans*-retinyl palmitate, 500,000 IU/g) 0.80, Vitamin D3 (cholecalciferol, 400,000 IU/g) 0.25, Vitamin K (phylloquinone) 0.075, HFD: high-fat diet.

MgPic, ZnPic, and SeMet were provided by Nutrition 21 LLC. (Harrison, NY, USA). MgPic, ZnPic and SeMet are composed of 9.052% magnesium, 21.126% zinc, and 40.265% selenium, respectively. The amount of elemental Mg, Zn, and Se were calculated based on 100, 30, and 0.1 mg Mg, Zn, and Se needed for a 70-kg adult human after adjusting doses based on metabolic body size(Nair and Jacob, 2016, Shin et al., 2010).

Bodyweight changes were recorded weekly. All rats were sacrificed by cervical dislocation at the end of the study. Blood samples were collected, and the visceral fat and organs (liver, kidneys, brain, pancreas, and heart) were removed from all animals from each group and weighted after sacrificing them. All samples (blood and organs) were stored at −80 °C until needed. The duration of the experiment was 8 weeks.

### 2.2 Laboratory analyses

Blood samples were centrifuged and used to analyze biochemical parameters. Serum parameters (glucose, total cholesterol, triglyceride, creatine, and blood urea nitrogen) and enzyme activities of aspartate aminotransaminase (AST) and alanine aminotransaminase (ALT) were measured using an automated analyzer (Samsung Electronics Co, Suwon, Korea). The serum insulin and leptin concentrations were determined with the Rat Insulin kits (Linco Research Inc, St. Charles, MO, USA) by ELISA (Bio-Tek Instruments Inc, Vermont, USA).

Insulin resistance index was assessed by homeostasis model assessment of insulin resistance (HOMA-IR) as (fasting glucose mg/dL) × (fasting insulin ulU/mL)/405. Because this calculation is human-based, basal concentrations are not the same in the rodents and should be re-estimated (strain differences) (Orhan, Kucuk, Tuzcu, Sahin, Komorowski, & Sahin, 2019). Hence, HOMA-IR was calculated with a formula adapted to Matthews et al. (Matthews, Hosker, Rudenski, Naylor, Treacher, & Turner, 1985). For male rats, reference values were calculated using average fasting glucose (82 mg/dL) and plasma insulin (18.6 ulU/mL) concentrations from all groups (56 rats) at the beginning of the study (day 0). The HOMA-IR score was calculated as the product of the fasting insulin level (ulU/mL) and the fasting glucose level (mg/dL), divided by 1525.2 for rats. The cut-off value to define insulin resistance was HOMA-IR ≥ 2.50. Rats presenting HOMA-IR ≥ 2.50 were considered insulin resistant.

Tissues were homogenized according to the previous method (Orhan et al., 2021) to determine the activities of superoxide dismutase (SOD), catalase (CAT), and glutathione peroxidase (GSH-Px) and malondialdehyde (MDA) levels. Based on the manufacturer’s procedure, the SOD, CAT, and GSH-Px activities were measured using ELISA kits (Cayman Chemical, Ann Arbor, MI, USA). To determine MDA levels, a High-Performance Liquid Chromatography (HPLC) apparatus of UV–vis SPD-10 AVP detector, a CTO-10 AS VP column, and 30 mM KH2PO4 and methanol (82.5: 17.5, v/v, pH 3.6) at a flow rate of 1.2 ml/min were used (Shimadzu, Japan).

Serum testosterone, FSH, LH, and SHBG concentrations were determined with an ELISA (Elx-800, Bio-Tek Instruments Inc, Vermont, USA) using kits specific to rats (Cayman Chemical Co., Ann Arbor, MI, USA) according to the manufacturer's instructions.

### 2.3 Atomic absorption spectrometry

The diet samples were dried in an oven at 60 °C and then ground for the analyses. About 0.3 g of sample was mineralized in HNO_3_ (5 ml) using a microwave digestive system (Berghof, Eningen, Germany). The heating program employed as described in the oven’s user manual. The samples were diluted with deionized water, and the mineral levels were determined. Levels of Mg and Zn in the diet samples were determined in triplicate by atomic absorption spectrometry (AAS, Perkin Elmer, Norwalk, CT, USA) with flame atomization in an acetylene-air via recognized and fully confirmed procedures at the following wavelengths: 285.2 nm for Mg and 213.9 nm for Zn with the Zeeman background correction. Se concentrations in the diets were measured by graphite furnace atomic absorption spectrometry using the same atomic absorption spectrometry with longitudinal Zeeman background correction and an AS furnace autosampler as previously described (Finley, Matthys, Shuler, & Korynta, 1996). The method was verified with certified reference materials (NIST SRM 1849a for Mg and bovine muscle BCR 184 for Zn and Se). All values were within the certified value range. The method's accuracy was 99.28, 99.37 and 99.46% for Mg, Zn, and Se. The limit detections were 0.057 and 0.065 mg/L and 1.79 µg/L for Mg, Zn, and Se, respectively.

### 2.4 Western blot analyses

The protein abundances of the liver SREBP-1c, LXRα, ACLY, FAS, NF-κB p65 (nuclear and phosphorylated) and Nrf2 were determined by Western blot. For this purpose, protein extraction was done by standardizing the liver in 1 ml of the ice-cold hypotonic buffer as described previously (Tuzcu, Orhan, Sahin, Juturu, & Sahin, 2017). Aliquots containing 20 μg of protein were subjected to 10% sodium dodecyl sulfate–polyacrylamide gel electrophoresis (SDS-PAGE) and transferred to nitrocellulose membranes for 1 h prior to application of primary antibodies. SREBP-1c, LXRα, ACLY, FAS, NF-κB and Nrf2 antibodies were diluted at 1:1000 in the buffer. Protein loading was controlled via an antibody against β-actin, and the bands were evaluated by an imaging system (NIH, Bethesda, USA).

### 2.5 Histopathology

Liver samples were fixed in 10% buffered formalin and processed for histopathologic analyses. 4 μm sections were cut, deparaffinized, dehydrated, and stained with hematoxylin and eosin (H & E) for light microscopy examination.

### 2.6 Statistical analyses

The sample size was calculated by the G*Power program (Version 3.1.9.2) with alpha error 0.05 and 85% power. Conformism to normality was implemented using the “Shapiro-Wilk” test, and the homogeneity of the variances was checked with the “Levene” test. The data were examined by one-way ANOVA (SPSS, 2012 Version 21.0), followed by the Tukey *post hoc* test. *P* < 0.05 was considered statistically significant.

## 3 Results and discussion

Since initial body weights were similar among treatments, final body weights reflected the more significant bodyweight gains as expected, with feeding an HFD (*P* < 0.05; Table 2). However, supplementing each mineral equally reduced the final body weights of the rats. Feeding an HFD compared with the control diet to the rats increased the visceral fat by 279% (*P* < 0.05). The visceral fat was mostly decreased by treating the combination of MgPic + ZnPic + SeMet with a lesser extent of other mineral supplements except for HFD + SeMet, which had similar values of HFD (*P* < 0.05). These results indicated that feeding an HFD causes overweight/obesity along with a greater body fat accumulation. Similar to the present work results, Maejima et al. (Maejima, Yokota, Horita, & Shimomura, 2020) found greater body weights and greater visceral and subcutaneous fat accumulation (30 vs 18% total body fat) in adult rats fed an HFD compared with adult rats fed a regular chow diet. Being parallel with the result of the present work, although not statistically significant, in terms of dietary mineral supplementation, supplementing 90 mg/kg zinc to the diet of mice was also shown to reduce body fat with reduced diameter and area of adipocytes (Qi et al., 2020).

**Table 2:** Effects of dietary supplementation of magnesium picolinate (MgPic), zinc picolinate (ZnPic) and selenomethionine (SeMet) on body, visceral fat and organ weights in rats fed a high-fat diet.

Items	Control	HFD	HFD + MgPic	HFD + ZnPic	HFD + SeMet	HFD + MgPic + ZnPic	HFD + MgPic + SeMet	HFD + MgPic + ZnPic + SeMet
Final BW (g)	317.71 ± 12.88^c^	426.00 ± 13.97^a^	391.43 ± 14.01^b^	389.00 ± 10.00^b^	410.71 ± 8.62^ab^	382.57 ± 7.36^b^	382.43 ± 5.85^b^	389.43 ± 8.87^b^
Visceral fat(g)	1.04 ± 0.06^c^	3.94 ± 0.44^a^	3.36 ± 0.25^ab^	3.11 ± 0.21^ab^	3.83 ± 0.28^a^	3.26 ± 0.31^ab^	3.42 ± 0.22^ab^	2.96 ± 0.17^b^
Liver (g)	9.66 ± 0.36^c^	11.99 ± 0.45^a^	11.35 ± 0.45^ab^	10.85 ± 0.27^ab^	11.47 ± 0.28^ab^	11.10 ± 0.64^ab^	10.95 ± 0.25^ab^	10.72 ± 0.24^bc^
Kidney (g)	1.43 ± 0.08	1.66 ± 0.08	1.61 ± 0.03	1.48 ± 0.03	1.61 ± 0.06	1.53 ± 0.04	1.51 ± 0.05	1.49 ± 0.04
Brain (g)	1.98 ± 0.03	1.97 ± 0.05	1.95 ± 0.03	1.85 ± 0.05	1.88 ± 0.05	1.87 ± 0.03	1.89 ± 0.04	1.95 ± 0.09
Pancreas (g)	1.45 ± 0.08	1.65 ± 0.13	1.71 ± 0.13	1.66 ± 0.17	1.71 ± 0.15	1.57 ± 0.08	1.50 ± 0.07	1.50 ± 0.07
Heart (g)	1.30±±0.07	1.48 ± 0.07	1.33 ± 0.04	1.48 ± 0.08	1.48 ± 0.09	1.46 ± 0.09	1.33 ± 0.03	1.25 ± 0.04

Statistical comparisons are indicated with different superscript (a-f) in the same row (*P* < 0.05; ANOVA and Tukey’s post-hoc test). Mean values are demonstrated with ± standard deviations. Dietary treatments are; Control: no treatment, HFD: high-fat diet, HFD + MgPic: HFD supplemented with magnesium picolinate (MgPic), HFD + ZnPic: HFD supplemented with zinc picolinate (ZnPic), HFD + SeMet: HFD supplemented with selenium methionine (SeMet), HFD + MgPic + ZnPic: HFD supplemented with MgPic and ZnPic, HFD + MgPic + SeMet: HFD supplemented with MgPic and SeMet, HDF + MgPic + ZnPic + SeMet: HFD supplemented with MgPic, ZnPic, and SeMet.

All measured internal organs, but the liver weights remained unchanged with dietary treatments (*P* > 0.05). The liver weights increased with feeding an HFD but supplementing organic minerals to the HFD ameliorated, with the same magnitude, the negative effect of feeding HFD (*P* < 0.05). However, although not significantly, feeding HFD + MgPic + ZnPic + SeMet provided the lightest liver weights next to those of control. Greater energy intake due to feeding HFD compared with that of control diet results in the accumulation of energy as fat in the body but also in liver cells leading to metabolic disorders such as obesity (Vernon, Baranova, & Younossi, 2011). Increased volume and mass of the liver were also stated by other researchers who fed rats ad libitum high-fat pellets compared with restricted intake of high-fat pellets (Wires, Trychta, Bäck, Sulima, Rice, & Harvey, 2017). Increased liver weights in rats fed an HFD followed the results of increased activities of ALT and AST, particularly ALT, which doubled, with feeding an HFD compared with feeding a control diet (*P* < 0.05; Table 3). Elevated ALT and AST enzyme activities may be considered as an indication of hepatocellular injury (Hall & Cash, 2012). However, supplementing minerals lowered the ALT and AST activities to certain degrees, with HFD + MgPic + ZnPic + SeMet most effective. Like the result of the present work, supplementing 90 mg/kg zinc to the diet of mice was found to promote liver function and protect the liver from injury by reducing AST and ALT enzyme activities (Qi et al., 2020).

**Table 3:** Effects of dietary supplementation of magnesium picolinate (MgPic), zinc picolinate (ZnPic), and selenomethionine (SeMet) on serum biochemical parameters in rats fed a high-fat diet.

Items	Control	HFD	HFD + MgPic	HFD + ZnPic	HFD + SeMet	HFD + MgPic + ZnPic	HFD + MgPic + SeMet	HFD + MgPic + ZnPic + SeMet
Glucose (mg/dL)	84.43 ± 1.94^e^	187.14 ± 5.65^a^	164.86 ± 3.46^bcd^	159.43 ± 6.11^bcd^	178.86 ± 5.29^ab^	152.57 ± 4.89 ^cd^	169.14 ± 3.78^abc^	147.71 ± 5.04^d^
Insulin (ulU/mL)	18.47 ± 0.39^e^	31.53 ± 0.32^a^	28.49 ± 0.33^bc^	26.33 ± 0.70 ^cd^	30.09 ± 0.75^ab^	25.73 ± 0.35^d^	28.09 ± 0.44^bc^	24.49 ± 0.48^d^
HOMA-IR	1.02 ± 0.10^e^	3.87 ± 0.28^a^	3.08 ± 0.13^c^	2.76 ± 0.43 ^cd^	3.53 ± 0.38^ab^	2.57 ± 0.16^d^	3.12 ± 0.24^bc^	2.36 ± 0.12^d^
TC (mg/dL)	82.74 ± 2.70^d^	123.14 ± 3.54^a^	106.46 ± 2.12^bc^	95.31 ± 2.64 ^cd^	116.06 ± 2.18^ab^	94.45 ± 4.84 ^cd^	103.77 ± 2.44^bc^	89.97 ± 1.39^d^
TG (mg/dL)	84.69 ± 1.42^c^	112.51 ± 1.27^a^	102.34 ± 1.47^ab^	93.43 ± 1.09^bc^	115.75 ± 4.62^a^	90.25 ± 5.21^bc^	97.43 ± 3.49^bc^	90.85 ± 2.13^bc^
Leptin (ng/mL)	20.93 ± 0.46^f^	39.18 ± 0.60^a^	31.74 ± 0.76^c^	30.14 ± 0.64 ^cd^	35.42 ± 0.65^b^	27.94 ± 0.74^de^	30.10 ± 1.07 ^cd^	26.04 ± 0.54^e^
AST (U/L)	135.00 ± 6.66^d^	176.86 ± 4.09^a^	161.29 ± 3.02^abc^	164.14 ± 3.00^ab^	157.86 ± 3.61^bc^	162.29 ± 1.63^abc^	152.71 ± 3.40^bcd^	144.57 ± 4.56 ^cd^
ALT(U/L)	78.43 ± 1.86^f^	147.29 ± 2.88^a^	103.29 ± 1.76 ^cd^	115.86 ± 3.77^bc^	117.71 ± 4.99^b^	99.29 ± 2.31^de^	101.57 ± 3.72^d^	86.28 ± 1.71^ef^
Creatine (mg/dL)	0.44 ± 0.02	0.54 ± 0.03	0.50 ± 0.03	0.47 ± 0.03	0.55 ± 0.03	0.48 ± 0.04	0.50 ± 0.02	0.47 ± 0.03
BUN (mg/dL)	20.29 ± 0.45	23.01 ± 0.99	23.09 ± 0.78	23.31 ± 0.73	21.83 ± 0.57	20.89 ± 1.24	22.54 ± 0.75	21.31 ± 0.87

TC: Total cholesterol; TG: Triglyceride; AST: Aspartate aminotransferase; ALT: Alanine aminotransferase. BUN: Blood Urea Nitrogen. HOMA-IR: homeostatic model assessment insulin resistance (rat), [HOMA-IR=(fasting glucose mg/dL) × (fasting insulin ulU/L)/1525.5]. Statistical comparisons are indicated with different superscript (a-f) in the same row (*P* < 0.05; ANOVA and Tukey’s post-hoc test). Mean values are demonstrated with ± standard deviations. Dietary treatments are; Control: no treatment, HFD: high-fat diet, HFD + MgPic: HFD supplemented with magnesium picolinate (MgPic), HFD + ZnPic: HFD supplemented with zinc picolinate (ZnPic), HFD + SeMet: HFD supplemented with selenium methionine (SeMet), HFD + MgPic + ZnPic: HFD supplemented with MgPic and ZnPic, HFD + MgPic + SeMet: HFD supplemented with MgPic and SeMet, HDF + MgPic + ZnPic + SeMet: HFD supplemented with MgPic, ZnPic, and SeMet.

Glucose and insulin serum concentrations as well as HOMA-IR increased when rats were fed an HFD instead of a control diet (*P* < 0.05; Table 3). However, as numerical values, supplementing organic minerals to the HFD, particularly MgPic + ZnPic + SeMet, decreased the HOMA-IR and glucose and insulin levels to certain degrees. In this respect, the treatments of HFD + MgPic + ZnPic and HFD + MgPic + ZnPic + SeMet were equally effective in reducing insulin serum levels. Since Zn contributes to the synthesis, storage, and secretion of insulin in pancreatic β-cells, Zn deficiency has been reported to decrease insulin sensitivity and glucose tolerance (Yang et al., 2015). Supplementing 90 mg/kg zinc to the diet of mice was found to promote the absorption of glucose and decrease the output of glucose along with increasing glycogen content in the liver (Qi et al., 2020).

Total cholesterol, triglycerides, and leptin concentrations in serum increased with feeding an HFD compared with a control diet (*P* < 0.05; Table 3). Supplementing organic minerals to the HFD, particularly HFD + MgPic + ZnPic + SeMet, however, decreased the levels of lipid parameters to certain degrees. More specifically, the HFD + MgPic + ZnPic + SeMet treatment was most effective in reducing the total cholesterol level close to that of control, whereas the HFD + MgPic + ZnPic + SeMet treatment provided the lowest leptin concentrations compared with that of HFD, but not being similar to that of control. Similar to the result of the present work in terms of dietary mineral supplementation, fecal triglyceride output and the expression of adipolysis genes were also found to increase in mice fed a diet supplemented with 90 mg/kg Zn (Qi et al., 2020).

Magnesium is involved in the etiology of many diseases, including cancer, stroke, asthma, cardiovascular problems, osteoporosis, immunity (de Baaij, Hoenderop, & Bindels, 2015) as well as diabetes, in which Mg concentrations were found low in patients with type 2 diabetes (Volpe, 2008). Being parallel to the results from the present study, rats with type 2 diabetes induced by feeding an HFD had increased serum insulin concentrations, insulin resistance (HOMA-IR), and triglycerides and total cholesterol concentrations when the diet was supplemented with MgCl_2_ at 250 mg/kg (Ige, Ajayi, & Adewoye, 2018). Similarly, rats fed an HFD had increased body weights, visceral fat, plasma insulin, leptin, and triglyceride concentrations, and supplementing Zn (6 mg/kg) to the HFD reduced the parameters (Thoen et al., 2019).

Co-supplementation of Mg-Zn and Mg-Se from the literature (Hamedifard et al., 2020, Zhang et al., 2018) also agreed with the present work results. Patients with type 2 diabetes and coronary heart diseases co-supplemented with 250 mg magnesium oxide and 150 mg zinc sulfate for 12 weeks had reduced plasma glucose, insulin, and HDL-cholesterol concentrations compared with a placebo group (Hamedifard et al., 2020). Also, rats fed an HFD co-supplemented with sodium selenite at 0.10 mg/kg body weight and magnesium gluconate at 58.33 mg/kg body weight were reported to reduce body weights, serum triglycerides, ALT and AST enzyme activities, and MDA levels compared with those of rats fed an HFD without supplementation (Zhang et al., 2018).

Varying results from the present work and the literature (de Baaij et al., 2015, Hamedifard et al., 2020, Ige et al., 2018, Qi et al., 2020, Thoen et al., 2019, Volpe, 2008, Zhang et al., 2018), in terms of significance and magnitude, could have been due to different forms and doses of trace minerals (Mg, Zn, Se, and their co-supplements) along with animal species used as models.

Feeding an HFD to rats resulted in increases in the serum, liver, and brain MDA concentrations, whereas decreases in the liver SOD, CAT, and GSH-Px concentrations (*P* < 0.05; Table 4). Supplementing the organic minerals to the HFD caused responses reversed, with HFD + MgPic + ZnPic + SeMet treatment being most effective. However, the treatments of HFD + SeMet alone, HFD + MgPic + SeMet, and HFD + MgPic + ZnPic + SeMet equally decreased the liver and brain MDA concentrations. Feeding an HFD to rats was also reported to increase MDA and decrease GSH levels (Cavaliere et al., 2018, Noeman et al., 2011). However, selenium-enriched yeast but not SeMet supplementation to healthy individuals decreased oxidative stress biomarkers (Richie et al., 2014). Mineral supplementations containing Se alone or in combinations would be expected to decrease MDA concentrations since Se is involved in antioxidant defense systems (Labunskyy, Hatfield, & Gladyshev, 2014). Selenium protects against cancer, heart problems, hyperlipidemia, and hyperglycemia (Vinceti et al., 2017, Wang et al., 2014). Tinkov et al. (Tinkov et al., 2020) also speculated that Se and selenoproteins might have crucial functions in adipose tissue physiology (Tinkov et al., 2020). However, the notion was not supported by the present work results in the sense that Se supplementation yielded similar serum cholesterol and triglyceride concentrations similar to those of HFD. Therefore, although Se has a strong antioxidant feature, its effects on diabetes and obesity have been inconclusive (Tinkov et al., 2020, Vinceti et al., 2015, Wang et al., 2016).

**Table 4:** Effects of dietary supplementation of magnesium picolinate (MgPic), zinc picolinate (ZnPic) and selenomethionine (SeMet) on antioxidative enzymes in rats fed a high-fat diet.

Items	Control	HFD	HFD + MgPic	HFD + ZnPic	HFD + SeMet	HFD + MgPic + ZnPic	HFD + MgPic + SeMet	HFD + MgPic + ZnPic + SeMet
Serum MDA (nmol/mL)	1.23 ± 0.04^d^	2.87 ± 0.09^a^	2.60 ± 0.07^a^	2.43 ± 0.11^ab^	1.82 ± 0.07^bcd^	2.41 ± 0.13^ab^	2.30 ± 0.32^abc^	1.70 ± 0.04 ^cd^
Liver MDA (nmol/g)	2.55 ± 0.16^c^	4.66 ± 0.32^a^	3.86 ± 0.13^ab^	3.60 ± 0.14^abc^	3.34 ± 0.50^bc^	3.72 ± 0.21^ab^	3.26 ± 0.12^bc^	3.19 ± 0.25^bc^
Brain MDA (nmol/g)	3.49 ± 0.15^d^	7.32 ± 0.23^a^	6.71 ± 0.13^ab^	6.26 ± 0.17^b^	5.14 ± 0.13^c^	6.51 ± 0.13^b^	5.27 ± 0.27^c^	5.18 ± 0.16^c^
Liver SOD (U/mg protein)	186.29 ± 2.56^a^	117.86 ± 2.67^f^	130.43 ± 2.71^e^	133.00 ± 2.99^de^	147.00 ± 1.59^c^	142.71 ± 2.36 ^cd^	152.14 ± 2.84^bc^	160.86 ± 2.03^b^
Liver CAT (U/mg protein)	290.43 ± 3.29^a^	213.36 ± 4.24^f^	231.29 ± 4.31^e^	243.4 ± 3.12^cde^	253.14 ± 4.51^bcd^	239.86 ± 3.72^de^	258.00 ± 2.29^bc^	261.86 ± 2.72^b^
Liver GSH-Px (U/mg protein)	37.43 ± 0.87^a^	15.71 ± 0.29^f^	19.00 ± 0.72^e^	19.14 ± 0.40^e^	23.29 ± 0.42 ^cd^	21.43 ± 0.65^de^	25.86 ± 0.34^c^	29.29 ± 0.68^b^

MDA: malondialdehyde; SOD: superoxide dismutase; CAT: catalase; GSH-Px: glutathione peroxidase. Statistical comparisons are indicated with different superscript (a-f) in the same row (*P* < 0.05; ANOVA and Tukey’s post-hoc test). Mean values are demonstrated with ± standard deviations. Dietary treatments are; Control: no treatment, HFD: high-fat diet, HFD + MgPic: HFD supplemented with magnesium picolinate (MgPic), HFD + ZnPic: HFD supplemented with zinc picolinate (ZnPic), HFD + SeMet: HFD supplemented with selenium methionine (SeMet), HFD + MgPic + ZnPic: HFD supplemented with MgPic and ZnPic, HFD + MgPic + SeMet: HFD supplemented with MgPic and SeMet, HDF + MgPic + ZnPic + SeMet: HFD supplemented with MgPic, ZnPic, and SeMet.

Serum testosterone, FSH, LH, SHBG, and IGF-1 hormone concentrations all decreased with feeding an HFD to rats compared with feeding a control diet (*P* < 0.05; Table 5). Supplementing minerals to the HFD resulted in increases in the hormone levels with HFD + MgPic + ZnPic + SeMet treatment, as numerical values are most effective. Also, the treatments of HFD + MgPic + ZnPic and HFD + MgPic + ZnPic + SeMet were equally effective in increasing testosterone levels. Similar to the present work results, reproductive dysfunctions measured as decreased semen quality, LH and testosterone concentrations, and degeneration in testes were reported in diabetic and obese rats compared with healthy rats (Abdel-Fadeil, Abd Allah, Iraqy, Elgamal, & Abdel-Ghani, 2019). Testosterone production is promoted by IGF-1 (Yoshizawa & Clemmons, 2000); therefore, decreases in IGF-1 concentrations would reduce the testosterone concentrations, as evidenced in the present work. Androgens and estrogens are regulated and transported to target tissues by SHBG (Avvakumov, Cherkasov, Muller, & Hammond, 2010). Therefore, male fertility measured as hormonal changes at the present work was negatively influenced by feeding an HFD to rats, and supplementing minerals to the HFD reversed the responses. In accord with the result from the current work, Khalaf et al. (Khalaf, Ahmed, Moselhy, Abdel-Halim, & Ibrahim, 2019) reported protective effects of sodium selenite at 3 mg/kg or nano selenium at 2 mg/kg against testicular toxicity induced by bisphenol A in male rats. In this respect, Se also has a role in fertility (Zhang, Wang, & Xu, 2008). Similarly, ZnO supplemented nanoforms at 3 mg per kg BW to male rats also protected against testicular toxicity and genotoxicity induced by doxorubicin (El-Maddawy & Abd El Naby, 2019). Also, rats with diabetic toxicity in testes had not only low plasma and testes concentrations of testosterone and 17 beta-estradiol but also reduced Mg concentrations (Hamden et al., 2008).

**Table 5:** Effects of dietary supplementation of magnesium picolinate (MgPic)0, zinc picolinate (ZnPic), and selenomethionine (SeMet) on serum concentrations of male reproductive hormones in rats fed a high-fat diet.

Items	Control	HFD	HFD + MgPic	HFD + ZnPic	HFD + SeMet	HFD + MgPic + ZnPic	HFD + MgPic + SeMet	HFD + MgPic + ZnPic + SeMet
Testosterone (ng/mL)	3.11 ± 0.06^a^	1.26 ± 0.05^e^	1.84 ± 0.05^d^	2.18 ± 0.05^c^	1.74 ± 0.04^d^	2.45 ± 0.02^b^	1.89 ± 0.04^d^	2.58 ± 0.03^b^
FSH (ng/mL)	0.59 ± 0.01^a^	0.16 ± 0.01^f^	0.31 ± 0.01^d^	0.41 ± 0.01^c^	0.22 ± 0.01^e^	0.38 ± 0.01^c^	0.33 ± 0.01^d^	0.49 ± 0.01^b^
LH (ng/mL)	1.59 ± 0.02^a^	0.63 ± 0.02^f^	0.83 ± 0.02^e^	1.05 ± 0.01^d^	0.74 ± 0.03^e^	1.15 ± 0.02^c^	1.03 ± 0.03^d^	1.28 ± 0.02^b^
SHBG (ng/mL)	0.65 ± 0.02^a^	0.19 ± 0.01^f^	0.27 ± 0.01^e^	0.31 ± 0.01^d^	0.21 ± 0.01^ef^	0.38 ± 0.01^c^	0.28 ± 0.01^d^	0.52 ± 0.02^b^
IGF-1 (µg/mL)	0.71 ± 0.01^a^	0.21 ± 0.01 ^g^	0.34 ± 0.01^e^	0.39 ± 0.01^d^	0.29 ± 0.01^f^	0.47 ± 0.01^c^	0.31 ± 0.01^ef^	0.55 ± 0.01^b^

FSH: Follicle-stimulating hormone; LH: Luteinizing hormone; SHBG: Sex hormone-binding globulin, IGF-1: Insulin-like growth factor 1. Statistical comparisons are indicated with different superscript (a-f) in the same row (*P* < 0.05; ANOVA and Tukey’s post-hoc test). Mean values are demonstrated with ± standard deviations. Dietary treatments are; Control: no treatment, HFD: high-fat diet, HFD + MgPic: HFD supplemented with magnesium picolinate (MgPic), HFD + ZnPic: HFD supplemented with zinc picolinate (ZnPic), HFD + SeMet: HFD supplemented with selenium methionine (SeMet), HFD + MgPic + ZnPic: HFD supplemented with MgPic and ZnPic, HFD + MgPic + SeMet: HFD supplemented with MgPic and SeMet, HDF + MgPic + ZnPic + SeMet: HFD supplemented with MgPic, ZnPic, and SeMet.

**Fig. 1:**
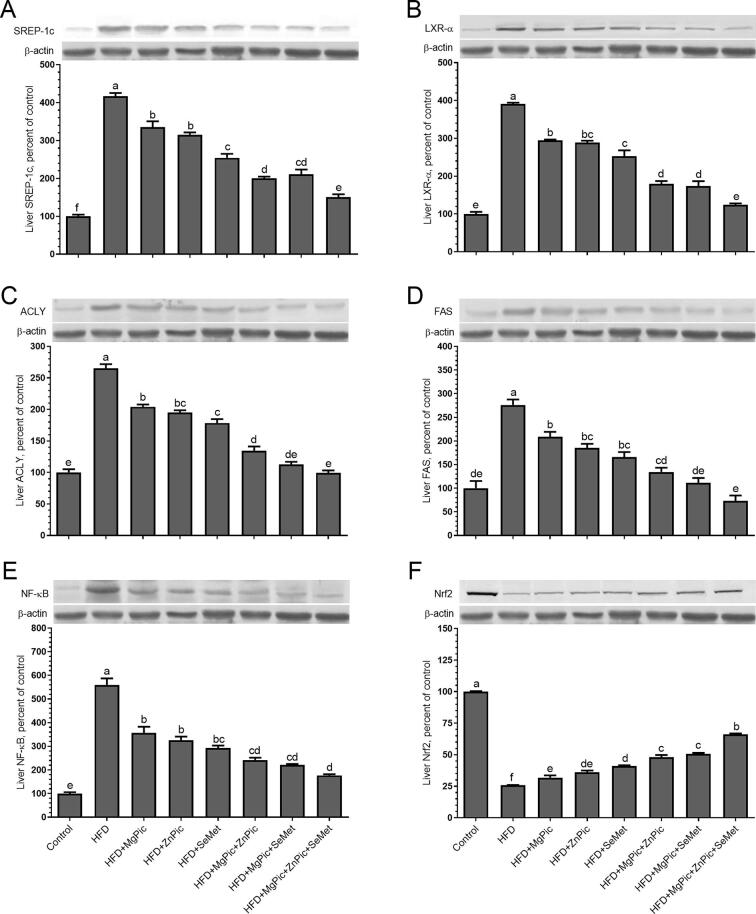
**Effects of dietary supplementation of magnesium picolinate (MgPic), zinc picolinate (ZnPic) and selenomethionine (SeMet) on liver level of SREP-1c, LXRα, ACLY, FAS, NF-κB and Nrf2 in rats fed a high-fat diet.** Dietary treatments are: Control: no treatment, HFD: high-fat diet, HFD + MgPic: HFD supplemented with magnesium picolinate (MgPic), HFD + ZnPic: HFD supplemented with zinc picolinate (ZnPic), HFD + SeMet: HFD supplemented with selenium methionine (SeMet), HFD + MgPic + ZnPic: HFD supplemented with MgPic and ZnPic, HFD + MgPic + SeMet: HFD supplemented with MgPic and SeMet, HDF + MgPic + ZnPic + SeMet: HFD supplemented with MgPic, ZnPic, and SeMet. Data are expressed as a ratio of the normal control value (set to 100%). Each bar represents the mean and standard error of the mean. The intensity of the bands shown in the band was quantified by densitometric analysis, and β-actin was included to ensure equal protein loading. Blots were repeated at least 3 times (n = 3). Different superscripts on top of each bar (A–E) indicate group means differences (*P* < 0.05).

Protein levels of liver SREBP-1c, LXRα, ACLY, FAS, and NF-κB increased, while Nrf2 levels decreased with feeding an HFD to rats compared with feeding a control diet (*P* < 0.05; Fig. 1). Supplementing minerals to HFD reversed the responses (protein levels) with various levels, HFD + MgPic + ZnPic + SeMet treatment being most prominent. More specifically, the levels of SREP-1c were equally decreased by HFD + MgPic alone and HFD + ZnPic alone, whereas LXRα level was equally decreased by HFD + MgPic + ZnPic and HFD + MgPic + SeMet treatments. Interestingly, the treatment of HFD + MgPic + ZnPic + SeMet provided similar values as the control for ACLY levels. The FAS levels were reduced with both HFD + MgPic alone and HFD + ZnPic alone treatments. Interestingly, the FAS levels were similar for the control and HFD + MgPic + SeMet treatments and even lower than those of control HFD + MgPic + ZnPic + SeMet treatment. The levels of NF-κB were equally decreased with HFD + MgPic alone and HFD + ZnPic alone but further and equally decreased by HFD + MgPic + ZnPic and HFD + MgPic + SeMet treatments. The treatments of HFD + MgPic + ZnPic and HFD + MgPic + SeMet were also equally increased the Nrf2 levels.

All protein levels were reversely influenced when HFD was increasingly supplemented with the minerals in HFD + MgPic, HFD + ZnPic, HFD + SeMet, HFD + MgPic + ZnPic, HFD + MgPic + SeMet, and HFD + MgPic + ZnPic + SeMet. However, the same order was not apparent with the other parameters measured. Similar to the results of the present work, mRNA expression levels of liver LXRα, SREBP-1c, and FAS increased in rats fed an HFD, and when the HDF was co-supplemented with sodium selenite at 0.10 mg/kg body weight and magnesium gluconate at 58.33 mg/kg body weight, the expression levels decreased (Zhang et al., 2018). Tuzcu et al. (Tuzcu, Orhan, Sahin, Juturu, & Sahin, 2017) also found greater hepatic protein levels of SREBP-1c, LXRs, ACLY, FAS, NF-κB, but lower Nrf2 expressions in rats fed an HFD. The results from the transcriptional factors revealed that feeding an HFD to rats increased lipid metabolism and oxidative stress by increasing the levels of SREBP-1c, LXRα, ACLY, FAS, and NF-κB which are known to promote lipogenesis, glucose-dependent fatty acid synthesis, and oxidative stress (Bai et al., 2019, Skrzep-Poloczek et al., 2020, Zhao et al., 2016). Supplementing organic elements, particularly the combination of all, to the HFD reversed the responses.

**Fig. 2:**
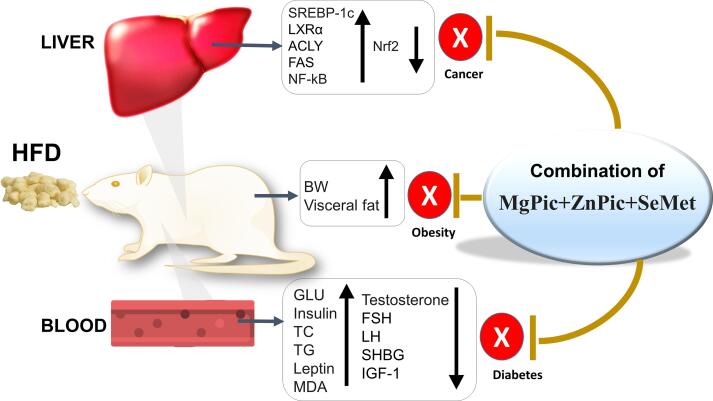
A schematic illustration of the effects of feeding HFD to rats and its consequences (analyzed parameters), and the mineral supplementation to reverse the negative effects in protecting obesity-related diseases.

Several transcription factors, including SREBP-1, LXRα, ACLY, FAS, NF-κB, and Nrf2 in regulating obesity, have been identified in the literature. SREBP-1c is involved in regulating fatty acid biosynthesis (Ferré, Phan, & Foufelle, 2021), whereas ACLY and FAS are responsible for *de novo* fatty acid synthesis (Pinkosky et al., 2017, Wang et al., 2004). As having a critical role in regulating cholesterol metabolism, LXRα plays a crucial part in atherosclerosis (Zhang et al., 2012). In addition, NF-κB p65 and Nrf2 regulate hepatic lipogenesis and lipid homeostasis (Daniel et al., 2021, Huang et al., 2010). The overexpression of liver SREBP-1c, which activates FAS and ACLY, causes increased visceral adipose tissue and hepatic lipid accumulation (Ito et al., 2013, Zhang et al., 2021). The activation of Nrf2 suppresses the transcription of LXRα and SREBP-1c (Fan et al., 2021). Changes in the expressions of the measured transcription factors at the present work with specific trace elements were against the lipid accumulation in hepatocytes and the development of obesity (Fig. 2).

**Fig. 3:**
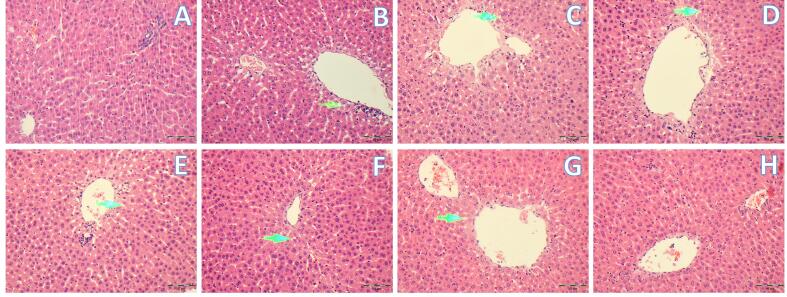
**Effects of dietary supplementation of magnesium picolinate (MgPic), zinc picolinate (ZnPic), and selenomethionine (SeMet) on histopathological changes as photomicrograph of liver sections in rats fed a high-fat diet.** The treatments are; **A**) Control (no treatment), **B**) high-fat diet (HFD), **C**) HFD supplemented with magnesium picolinate (MgPic) (HFD + MgPic), **D**) HFD supplemented with zinc picolinate (ZnPic) (HFD + ZnPic), **F**) HFD supplemented with selenium methionine (SeMet) (HFD + SeMet), **E**) HFD supplemented with MgPic and ZnPic (HFD + MgPic + ZnPic), **G**) HFD supplemented with MgPic and SeMet (HFD + MgPic + SeMet), or **H**) HFD supplemented with MgPic, and ZnPic as well as SeMet (HDF + MgPic + ZnPic + SeMet). H&E × 200.

Histopathological evidence from H&E staining suggests mild hepatic steatosis caused by feeding HFD compared to the control diet fed to rats (Fig. 3). Also, HE staining showed that the structure of the liver cells was destroyed, and a large number of fat vacuoles were observed. The HFD caused rat liver fat accumulation, and the lipid metabolism balance in the liver was damaged. However, the mineral supplementation particularly ameliorated the negative effects of feeding HFD. These results agreed with the results from the liver weights and ALT-AST enzyme activities of the present work.

The limitation of the present study should be acknowledged in a way that treatments did not contain an “HFD + ZnPic + SeMet” group which would give an idea of the effects HFD + ZnPic + SeMet combination before evaluating the combination of all organic minerals (HFD + MgPic + ZnPic + SeMet). It would also be interesting to see a possible effect(s) of a combination of HFD + ZnPic + SeMet treatment. Study results from dietary supplementations of a combination of organic Zn and organic Se in humans or animal models have been scarce in the literature. Mahmoodianfard et al. (Mahmoodianfard et al., 2015) found that overweight or obese female hypothyroid patients supplemented with Zn gluconate alone or a combination of selenium yeast and zinc gluconate for 12 weeks had both increased serum concentrations of free triiodothyronine (FT3). Although the combination of HFD + ZnPic + SeMet treatment would have been expected to have greater responses than those of each alone, it could be only speculation.

The results of the present work revealed that none of the organic elements alone or in the combination of the two exerted a prominent effect on parameters measured. More specifically, the HFD + SeMet treatment alone was ineffective in influencing the most measured parameters, yielding similar values to HFD. However, since Se is involved in the oxidative stress mechanism, the effect of Se was apparent only with oxidative stress-related parameters. The treatment of HFD + ZnPic alone or in a double combination yielded values similar to or close to HFD + MgPic + ZnPic + SeMet treatment in such parameters as insulin and hormone concentrations. Overall, although not additive or synergistic, the combination of all organic minerals added to HFD (HFD + MgPic + ZnPic + SeMet) provided the greatest responses. Therefore, supplementation of a combination of MgPic, ZnPic, and SeMet is recommended for protective effects in diabetes- and obesity-related problems in rats which may be a model for human cases.

## Funding

This research was financially supported by Nutrition 21, LLC, NY, USA, and the Turkish Academy of Science (Ankara, Turkey; KS, in part).

## CRediT authorship contribution statement

**Kazim Sahin:** Conceptualization, Supervision, Project administration, Funding acquisition, Writing – review & editing. **Cemal Orhan:** Methodology, Formal analysis, Investigation, Visualization, Validation. **Osman Kucuk:** Methodology, Writing – original draft. **Mehmet Tuzcu:** Methodology, Formal analysis, Investigation. **Nurhan Sahin:** Methodology, Formal analysis, Investigation. **Ibrahim H. Ozercan:** Methodology, Investigation. **Sarah Sylla**: Writing – original draft, Writing – review & editing. **Sara P. Ojalvo:** Writing – original draft, Writing – review & editing. **James R. Komorowski:** Writing – review & editing.

## Declaration of Competing Interest

James R. Komorowski, Sara P. Ojalvo, and Sarah Sylla are employed by Nutrition 21, LLC, NY, USA. The remaining authors declare that they have no known competing financial interests or personal relationships that could have appeared to influence the work reported in this paper.
